# Potentially toxic element bioaccumulation in consumed indoor shrimp farming associated with diet, water and sediment levels

**DOI:** 10.1007/s11356-023-30939-1

**Published:** 2023-11-14

**Authors:** José Joaquín Ramos-Miras, Maria Jose Sanchez-Muros, Patricio Renteria, Carlos Gil de Carrasco, Luis Roca-Perez, Mireia Boluda-Navarro, Javier Pro, Jose Antonio Rodríguez Martín

**Affiliations:** 1 Dpto. Didácticas Específicas, Universidad de Córdoba, Avda. San Alberto Magno s/n, 14071 Córdoba, Spain; 2 Dept. Biology, and Geology, University of Almería, Ctra. de Sacramento s/n, La Cañada, 04120 Almería, Spain; 3 Faculty of Agricultural Sciences, Technical University of Machala, 070102 Machala, Ecuador; 4 Dept. Biologia Vegetal, Facultat de Farmàcia, Universitat de València, Av. Vicent Andrés I Estellés S/n, 46100 Burjassot, Valencia Spain; 5 Healthincode, Calle de la Travesía s/n, 46024 Valencia, Spain; 6 Instituto Nacional de Investigación y Tecnología Agraria y Alimentaria (INIA-CSIC), Ctra. de la Coruña km. 7,5, 28040 Madrid, Spain

**Keywords:** Heavy metals, Shrimp farming, Food safety, Shrimp biometrics, Trace elements accumulation

## Abstract

Shrimp production is an important industry for many countries and shrimp consumption is increasing worldwide. Shrimps are a highly nutritional food, but can pose a risk for human health if subject to high levels of environmental contaminants. This work studies the presence of As, Cd, Co, Cr, Cu, Hg, Ni, Pb and Zn in shrimps from Ecuador and compares them to such contents noted in other shrimp-production areas in the world to evaluate the possible risks associated with these elements for consumer health, and to relate them to potentially toxic element (PTE) contents in water, sediments and diets, and also to animal biometric parameters. The PTE levels (mg kg^−1^ DM) obtained are as follows: in the head—As (3.52–6.11), Cd (0.02–0.10), Co (0.14–0.49) Cr (0.23–4.89), Cu (99.9––233.0), Ni (0.52–1.86), Pb (0.24–1.09), Zn (51.8–100.5) and Hg (μg kg^−1^ DM) (10.00–66.81); in the tail—(0.91–3.21), Cd (0.01–0.02), Co (0.01–0.43) Cr (0.01–6.52), Cu (20.0–72.44), Ni (0.15–2.03), Pb (0.01–0.69), Zn (31.2–66.1) and Hg (μg kg^−1^ DM) (10.00–67.18). The concentration of all the PTEs is generally lower than the limits set for seafood by European regulations, except for As in the cephalothorax (4.63 mg kg^−1^). Different behaviours for PTE accumulation in shrimps were found, which preferentially tend to accumulate in the cephalothorax, except for Hg (40.13 μg kg^−1^ DM), which accumulates in muscle (body) and is associated with contents of proteins, lipids and total shrimp weight. Nonetheless, the target hazard quotient (THQ) values for PTEs indicate that the consumption of shrimp muscles from Ecuador does not pose a human health risk because the values of these indices are below 1 in all cases.

**Supplementary Information:**

The online version contains supplementary material available at 10.1007/s11356-023-30939-1.

## Introduction

Shrimps have become one of the most eaten seafood species worldwide (Arisekar et al. 2022) and are an excellent source of proteins and also have low saturated fat contents, which make them a very health food option (Dayal et al. 2013). Shrimp aquaculture is generally perceived as less harmful to the environment than other forms of agriculture, urban development and industrialisation (Páez-Osuna 2001). Indoor shrimp farming is a rapidly growing industry, but has the potential to accumulate potentially toxic elements (PTEs) in shrimp tissues, which can pose a human health risk. PTEs are chemical elements that can be toxic at low concentrations and can accumulate in the food chain. Hg, Cd, Pb, As and Cr are some of the commonly studied PTEs in shrimp farming. PTE levels in indoor shrimp farming can be influenced by several factors, including shrimps’ diet, water quality and sediments. Shrimp feed can be a source of PTEs, particularly if made with contaminated ingredients. Water quality is also important because PTEs can be present in the water source and shrimps can absorb them through their gills. Sediment levels can also contribute to PTE accumulation because shrimps can ingest sediment particles that contain PTEs (Hidayati et al. 2020).

PTE bioaccumulation in shrimp tissues can vary depending on the type and level of exposure. PTEs can accumulate in both shrimp head and body tissues, and their levels can be influenced by shrimps’ biometric parameters, such as size and age. Larger shrimps usually tend to have higher PTE levels because they have more time to accumulate them. However, the relation between biometric parameters and PTE accumulation can vary depending on the specific PTEs and other environmental factors. It is important to monitor PTEs levels in indoor shrimp farming to ensure that they fall within the safe limits for human consumption. Regular testing of water, sediment and shrimp tissue samples can help to identify potential risks and inform management strategies to reduce PTE accumulation. Additionally, selecting high-quality feed ingredients and maintaining good water quality can help to minimise PTE exposure in shrimp farming. Contaminants like Hg, Cd, Pb and As can accumulate in various shrimp tissues, including the hepatopancreas (digestive gland), gills, muscle and the exoskeleton. The accumulation pattern of heavy metals in shrimp tissues can vary depending on the type of metal, exposure route, and exposure duration and intensity. In general, the hepatopancreas tends to accumulate higher heavy metal levels compared to other shrimp tissues. This is because the hepatopancreas is the primary site of metal detoxification and storage in shrimp. Gills can also accumulate high heavy metal levels because they are in direct contact with the surrounding water and can absorb metals through their surface (Ali and Khan 2019). Shrimp muscle tissue is commonly consumed by humans and, thus, heavy metal accumulation in this tissue is a particular food safety concern. Heavy metals can accumulate in shrimp muscle tissue if they are present in the surrounding water and/or diet. Therefore, it is important to monitor heavy metal levels in both the water and feed used in shrimp farming to ensure that their levels fall within the safe limits for human consumption. Overall, heavy metal accumulation in shrimp tissues can pose a health risk to the humans who consume them. Regular monitoring and management of heavy metal levels on shrimp farms can help to reduce this risk and to ensure the safety of shrimps as a food source.

Shrimp production in Ecuador is an extremely important industry for the economy of this country and is located along some 2237 km of its coastline, which is proportionally less territory than Mexico and Brazil. Ecuador produces more than 50% of the shrimps farmed in the western hemisphere (Monsalve and Quiroga 2022). In 2015, Ecuador’s shrimp production exceeded 300,000 tn (Uzcátegui et al. 2016) and it was second shrimp-exporting country (Argandona 2016). In 2021, Ecuador led the list of the major shrimp-producing countries, followed by China, Vietnam, India and Indonesia. In 2021, Ecuador’s shrimp sector’s deliveries amounted to $4539 million, which was 34% more than in 2020. The main importers of shrimps from Ecuador are the USA, China, the European Union and Japan (EUMOFA 2018; NationalMarineFisheriesService 2017). The main shrimp farming in Ecuador takes place along the Guayas Province and the majority of its shrimp production derives from semi-extensive shrimp farming in large ponds (1–10 ha) that lie on natural substrate and in water from rivers, estuaries and coasts. The El Oro Province has vast mining resources of is gold deposits (Mestanza-Ramón et al. 2022; Escobar-Segovia et al. 2021) with a high percentage of illegal exploitations that generate a volume of uncontrolled waste. Such waste affects the water bodies and soils in the area (Ramírez and Lacasaña 2001). Mining activities can result in the release of PTEs to the environment, which can have harmful effects on the ecosystem (Nanos et al. 2015; Odumo et al. 2018; Zamani-Ahmadmahmoodi et al. 2020; Mirzaei et al. 2014; Rodríguez Martín et al. 2014). Such mining discharges reach surface water bodies and accumulate in sediments (Odumo et al. 2014; Zamani-Ahmadmahmoodi et al. 2020). In our case, this situation affects water resources like River Chaguana, River Puyango, River Siete, River Gala and River Chico, and generally the water system in the area (Appleton et al. 2001) that, in turn, affects the El Oro Province region (Vilela-Pincay et al. 2020). This contamination of water resources implies the accumulation of contaminants by aquatic organisms directly absorbed from water, and indirectly through food chains (Łuczyńska et al. 2018) in relation to food (Livingstone 2001). Common mechanisms exist in metal toxicity, such as interactions with sulphydryl groups, essential metals and oxidative stress (Frías-Espericueta et al. 2022).

In short, PTEs are a concern for shrimp farming because they can accumulate in shrimp tissues and can potentially pose health risks to the humans who consume them. Heavy metals in shrimp farming are frequently related to the water and feed content (Arisekar et al. 2022; Batvari et al. 2016; Biswas et al. 2021; Dadar et al. 2014). Heavy metals, such as Pb, Hg and Cd, can enter water. To minimise the risk of heavy metal contamination in shrimp farming, it is important to accurately evaluate the risks of these PTEs on shrimp farms. The objectives of this work are to (i) evaluate the levels of potentially toxic elements (PTEs; As, Cd, Co, Cr, Cu, Hg, Ni, Pb, Zn) in Pacific White shrimp (*Litopenaeus vannamei*), and in both the tail and head; (ii) assess the environment in which they are farmed (water, sediment and food); (iii) study if PTE accumulation exists in Pacific White shrimp under such conditions; and (iv) evaluate the potential risk of these elements in shrimps for the health of those who eat them.

## Material and methods

### Shrimp-farming area and pollution sources

The shrimp-farming area is localised in the El Oro province of Ecuador (3° 23′ 45″ S; 79° 57′ 21″ W). El Oro is a coastal province in Ecuador known for its shrimp-farming industry. The region has several shrimp farms that produce large quantities of shrimps. It faces several challenges due to pollution from various sources. One of the primary sources of pollution to affect the El Oro shrimp farming is agricultural runoff. The region is known for its intensive agricultural practices, including the use of fertilisers and pesticides. These chemicals can leach into nearby waterways and eventually make their way to shrimp ponds, where they can harm shrimps and reduce production. Industrial pollution is another source of pollution that affects shrimp farming in El Oro. The province has several industries, including mining, which can release pollutants to water.

Historically, this province has been an important gold-mining centre, hence the name “Oro,” meaning “gold” in Spanish. The most important gold-mining areas in the El Oro Province are in the Nambija and Buenos Aires regions. Nambija, located near the city of Zamora, is known for its alluvial gold deposits. Buenos Aires, located near the city of Machala, has both alluvial and underground gold deposits. In the twentieth century, large-scale mining operations began to develop in the province, particularly in the Zaruma and Portovelo areas. These mines primarily extracted gold and silver, and contributed significantly to the local economy. Today, mining in El Oro is mostly small-scale and artisanal, with many informal mining operations scattered throughout the province (Mestanza-Ramón et al. 2022). Other minerals that are mined in Oro Province include Cu, Au and Pb. In recent years, the Ecuadorian government has sought to regulate and formalise the mining sector in El Oro. These pollutants can contaminate the environment and affect the health of shrimps and other aquatic organisms.

### Shrimps, sediment and water sampling

Shrimps were randomly distributed and stocked in eight 1-m^3^ cages placed inside a 1-ha pond on the “Noblecilla Salas” shrimp farm located in Santa Rosa (El Oro Province, Ecuador). Shrimps were fed 3 times daily (7% of their body weight) at 08:00 h, 12:00 h and 16:00 h. Every week, the total weight of shrimps was recorded to adjust the daily amount of feed. The water quality parameters were measured once a day and kept optimal for white shrimps’ growth and survival: average temperature (26.6°C), salinity (24.5‰) and pH (7.19). The proximal composition in the shrimp diet is shown in Table 1 (moisture, ash, lipid, protein and carbohydrate contents) and biometric parameters in Table S1. These food components may be of interest in the food industry for product development, quality control (QC) or regulatory purposes. Neutral detergent fibre (NDF) primarily contains major cell wall components, including hemicellulose, cellulose and lignin. Nitrogen-free extract (NFE) is designed to provide an estimate of water-soluble polysaccharides (sugars, starch) and is calculated by the difference between the original sample weight and the sum of the weights of moisture (water), ether extract, crude protein, crude fibre and ash. At the end of the feeding trial, shrimps were sacrificed, and head and abdomen were separated and frozen at −20°C until mineral determinations were made.

**Table 1:** Proximate composition

	Mean	SD
Proximate composition		
Crude protein	30.1	0.1
Total lipid	7.2	0.2
Ash	7.1	0.2
Nitrogen-free extract (NFE)	39.7	0.5
Neutral detergent fibre (NDF)	14.8	2.7

Proximate composition in % on dry matter basis

The water samples were packed in 4-L glass bottles. Two composite water samples were taken at the supply point of the pond by applying Regulation AM 097A of the Ministry of the Environment of Ecuador. Each sample was labelled and frozen at −20°C for preservation purposes and the subsequent PTE analysis. Sediment samples were collected from the bottom of the shrimp pool using a 2-inch PVC pipe at a depth of 20 cm. Samples were taken in a zig-zag pattern in an attempt to cover the shrimp-farming pool area. In the laboratory, the sediment samples were dried in an oven at 40°C. Samples were then pulverised through a 90–100-μm sieve before sieving.

### Analytical methods of potentially toxic elements

The Pacific Whitelegs shrimp (*Litopenaeus vannamei*) samples (head and body tissues) were digested by the *aqua regia* technique using microwave acid digestion (ETHOS SEL Model Milestone, Monroe, CT, USA). The concentrations of PTEs (As, Cd, Co, Cr, Cu, Ni and Pb) in tissue extracts were determined by atomic absorption spectrometry (PerkinElmer, Shelton, CT, USA 06484-4794) using graphite furnace atomic absorption spectrophotometry (GF-AAS) equipment. The limits of detection (LoDs) were 0.05 mg kg^−1^ for As, 0.002 mg kg^−1^ for Cd, 0.2 mg kg^−1^ for Co, 0.2 mg kg^−1^ for Cr, 0.014 mg kg^−1^ for Cu, 0.07mg kg^−1^ for Ni, 0.05mg kg^−1^ for Pb and 0.05 mg kg^−1^ for Zn. To check the accuracy and precision of measurements, PTE analyses were performed using two certified reference materials, CRM 463 and ERMI-CE278, from European Reference Materials. Recoveries for shrimp samples were good, and averaged between 95% for Cu and 104% for As. The same methodology was followed to determine PTEs in the sediment samples BCR-141 R and was used as the certified reference material to verify the method’s accuracy, which resulted in almost complete PTEs recovery (between 94% for Cu and 101% for As). Three replicates were analysed per sample and concentrations were indicated as mg kg^−1^ dry matter (DM).

The total Hg in the sediment, water and shrimp (head and body) was measured using a direct Hg analyser (DMA80, atomic absorption spectrophotometer, Milestone, Wesleyan University, Middletown, CT, USA). To determine the method’s precision, three replicates of each sample were considered. DMA80 provides two working ranges for Hg detection: 0–40 and 40–400 ng. The LoD was 0.5. The analytical procedure validation of the soil and sediment samples was performed with calcareous loam soil (BCR-141 R) and river sediment (BCR-320) obtained from the European Commission Community Bureau of Reference (ECCBR). The Hg analysis revealed a good agreement between the obtained and certified values, with an average recovery of 97.25% (2.1% VC). Three replicates were also analysed per sample and concentrations were expressed as mg kg^−1^ DM.

### Bioaccumulation and toxicity indices

#### Sediment and diet accumulation factors (BSAF and BDAF)

Studying PTEs in the natural environment is important for their toxicity, persistence and bioaccumulation there. It is known that several authors have employed bioaccumulation indices to estimate the accumulation of contaminants in different species of living beings and their tissues, and to relate them to their environment and their transfer from the abiotic environment, which could lead to their biomagnification (Silva et al. 2018; Avigliano et al. 2019; Xu et al. 2019). These authors have employed the biota sediment accumulation factor (BSAF) by assuming that the organism and sediment are in equilibrium (Mackay et al. 2018)

$$BSAF=\frac{Ct}{Cs}$$

where *Ct* is the concentration of the considered element in the species’ tissue (in our case, shrimp head and body), expressed as mg kg^−1^wet weight (WW) and *Cs* is the concentration of the same element in sediment, expressed as its mean value (mg kg^−1^DM).

As the studied species has been fed a diet that has been characterised, this index was contemplated in relation to the supplied diet (BDAF), and was calculated following the same criterion as BSAF.

$$BDAF=\frac{Ct}{Ci}$$

where *Ct* is the concentration of the considered element in shrimp tissue (mg kg^−1^ WW) and *Ci* is the concentration of this same element in the supplied diet, expressed as its mean value (mg kg^−1^DM).

BSAF or BADF values over 1 indicate that the living being or its tissue accumulate the considered element, and values over 2 represent super-concentrating tissues (Avigliano et al. 2019). Ali and Khan (2019) indicate that the BASF index would be a good indicator of the trophic transfer of heavy metals

##### Target hazard quotient

To calculate the possible non-carcinogenic health risk for the population exposed to any contaminants or toxins present in food, several authors have proposed using the target hazard quotient (THQ) index (Copat et al. 2018; Ramos-Miras et al. 2019; Traina et al. 2019; Yu et al. 2020; Dietrich and Ayers 2021; Jiao et al. 2021; Arisekar et al. 2022) .

For every contaminant, the THQ was calculated by considering the adult individuals with this formula:

1 $$THQ=\frac{\left( EF\times ED\times IR\times C\right)}{\left( RfD\times BW\times AT\right)}\times {10}^{-3}$$

where:

(i) EF represents exposure frequency (in our case, 70 years)
(ii) ED is the exposure duration (70 years)
(iii) IR is the ingestion rate (g day^−1^), which indicates the quantity of shrimps consumed per year. Based on the information in the bibliography, the mean consumption in 2016 for Europe was 1.56 kg/person and year (EUMOFA 2018) and 2 kg/person and year for the USA (NationalMarineFisheriesService 2017). Placing more importance in the EU, where 38% of shrimps were imported from Ecuador as opposed to 11% to the USA, makes Ecuador the third shrimp importer in volume terms in the EU (EUMOFA 2018). In the USA, 11% of consumed shrimps come from Ecuador and the mean consumption in 2017 was approximately 2 kg/person a year (NationalMarineFisheriesService 2017).
(iv) *C* is the concentration of the contaminant in shrimp tissue, expressed as wet weight (mgkg^−1^ WW).
(v) RfD is the oral reference dose (μg g^−1^ day^−1^) set by the Environmental Protection Agency of the USA (USEPA 2000).
(vi) BW is mean weight of adults (kg). For this calculation, the mean weight of adult citizens is considered, taken as 80.7 kg in the USA and as 70.8 kg in Europe (Walpole et al. 2012).
(vii) AT represents the averaging time (AT = EF × LT, where LT denotes individuals’ lifetime mean for about 70 years).

For THQ index calculation purposes, the analysed Hg was taken as MeHg (methyl mercury) (García et al. 2016), and only 3% of the total As was considered to be inorganic (Copat et al. 2018). Calculating the index was limited to shrimp bodies because it is the main shrimp part that is eaten.

As exposure generally occurs with more than one toxin, and as toxins can have additive effects, the hazard index (HI) was calculated as the arithmetic sum of the different THQs (THQ_i_) as so:

2 $$HI=\sum\nolimits_{i=1}^n{THQ}_i$$

If the obtained HI value exceeds 1, consuming the studied shrimps should be restricted because it poses a consumer health risk.

### Multivariate statistical analysis

Classic statistics (mean, median, coefficient of variation, standard deviation, etc.) was carried out to evaluate PTEs contents (As, Cd, Co, Cr, Cu, Ni, Pb and Zn) in both the Pacific Whitelegs shrimp (head and body tissues) and the medium in which they are farmed (water and sediments). An analysis of variance (ANOVA) was used to explore the effects of shrimps and tissues on the evaluated PTE contents. The multiway analysis of variance model included the main effects (tissues and sediments or water) and also the interactions between the main effects (tissue × diet). However, these classic statistical approaches ignore the relations between groups of variables. One approach to study the relations between the two sets of variables is to use the canonical correlation analysis (CCorA), which describes the relation between PTEs in shrimp (head and body tissues) and the shrimp biometrics (size and analytical body composition). The CCorA is a multivariate analysis of correlation in which one set of variables is not necessarily independent and the other is dependent, although that may potentially be the approach. This method is used considerably in ecology and, unlike the redundancy analysis (RDA), this method is symmetrical. With Y1 and Y2 (PTEs contents in head and body), and response variables (Y2 based on the shrimp biometric indices and body composition parameters), with variables *p* and *q*, respectively, we obtain:

3 $$\rho (i)= cor\left(Y1a(i),Y2b(i)\right)=\frac{\mathit{\operatorname{cov}}\left(Y1a(i),Y2b(i)\right)}{\mathit{\operatorname{var}}\left(Y1a(i)\right),\mathit{\operatorname{var}}\left(Y2b(i)\right)}$$

The CCorA provides two vectors, *a*(*i*) and *b*(*i*), which are maximised. Constraints must be introduced so that the solution for *a*(*i*) and *b*(*i*) is unique because the ultimate intention is to maximise the covariance between Y1*a*(i) and Y2*b*(*i*) and to minimise their respective variance (Jobson 1992; Takoutsing et al. 2018). The CCorA results are presented as graphical bi-plot scaling to evaluate the relation between biological variability and sensitivity to chemical disturbance (Losi et al. 2013; Campos-Herrera et al. 2016; Takoutsing et al. 2017). All the statistical analyses were carried out by the XLSTAT (Addinsoft Version, 2012.2.02) package for Windows.

## Results and discussion

### PTE level in shrimp farming associated with diet, water and sediment

The PTE concentration in the water, diet and sediments in the studied fish-farming ponds appear in Table 2. The PTE levels found in water were below the LoD, and only As was detected, but at very low concentrations (0.03 mg l^−1^). A priori, this finding suggests that the environment in which shrimps are farmed is adequate and, if PTEs possibly appeared in ponds, it would not be in a soluble form. Gills are the first target of water pollutants because they are constantly in contact with the external environment, although the epipodites of *L. vannamei* are more susceptible to the mixture of metals than gills (Frías-Espericueta et al. 2022).

**Table 2:** PTE in diet, water and sediment of shrimp farming

PTE	Water (mg l^−1^)	Sediment (mg kg^−1^DM)	Shrimp diet (mg kg^−1^DM)
As	0.03±0.02	131.4±8.8	2.68±0.13
Cd	b.d	0.51±0.06	0.32±0.04
Co	b.d	17.24±1.51	0.72±0.15
Cr	b.d	33.18±1.99	2.10±0.45
Cu	b.d	20.95±1.54	107.97±12.56
Hg	b.d	31.43±13.40	19.07±3.36
Ni	b.d	8.66±0.67	6.96±0.86
Pb	b.d	34.09±2.33	1.03±0.22
Zn	b.d	77.66±6.31	727.68±62.45

PTE contents in mg kg^−1^ except Hg in μg kg^−1^

As expected, the sediments in the shrimp ponds had a significantly higher percentage. These sediments play a fundamental role as indicators of contamination in marine ecosystems (Caballero-Gallardo et al. 2020). One of the environmental impacts of shrimp aquaculture is the accumulation of nutrients and other chemicals in sediments (Páez-Osuna 2001). In fact the toxicological effect of the metals associated with sediments in water environments is contemplated in the Sediment Quality Guidelines (SQG) (Table S2) for the protection and management of water ecosystems (MacDonald et al. 2000; Lopes et al. 2018; Caballero-Gallardo et al. 2020). The SQG sets three levels: threshold effects level (TEL), defined as the level below which there are no negative effects on aquatic fauna; probable effect level (PEL), which represents the concentration over which the frequency at which adverse effects on organisms is expected to occur; effects range medium (ERM), which designates the level over which toxic effects are generally expected to occur on aquatic living beings like low species richness in benthic communities or chronic toxicity. The concentration of Cd (0.51 mg kg^−1^), Cr (33.18 mg kg^−1^) and Hg (0.03 mg kg^−1^) in sediments in shrimp ponds (Table 2) was below the TEL (Table S2) and obtained similar levels to, or often lower levels than other shrimp farms elsewhere in the world, such as Bangladesh (Dietrich and Ayers 2021), Indonesia (Hidayati et al. 2020), Hong Kong (Cheung and Wong 2006) or India (Guhathakurta and Kaviraj 2000). Likewise, the contents of Cu (20.95 mg kg^−1^) and Pb (34.09 mg kg^−1^) observed in sediments were slightly over the TEL (Table S2). The main source of Cu and Zn metals in aquiculture systems was diet because Cu and Zn are added to shrimp diet as a mineral supplement (León-Cañedo et al. 2017). In line with this, we found that the contents of Cu (107.99 mg kg^−1^) and Zn (727.68 mg kg^−1^) in diet (Table 2) were 5–10-fold higher than those in sediments. Nevertheless, the concentration of heavy metals in shrimps’ food was lower than the maximum residual limits (MRLs), which are regulated by European legislation for animal food, except for Zn, which was over the MRLs (582 mg kg^−1^ WW *vs.* 150 mg kg^−1^ EU) (EC-EuropeanCommission 2015, 2002).

Nonetheless, the As levels found in these sediments (mean 131.4 mg kg^−1^) lead to the greatest concern because they are 10-fold higher than those reported by other studies (Dietrich and Ayers 2021) and they also exceed the MRLs (70 mg kg^−1^) according to the SQG criterion, which could lead to possible toxicity problems for shrimps and their consequent effect on the humans who eat Ecuadoran aquaculture products These levels suggest that As in sediments are of anthropogenic origin and might be derived from mining. The presence of As in soils and sediments is the result of the different physico-chemical factors that allow this metalloid to be leached, transported and retained, and are closely linked with geological characteristics. As previously mentioned, the El Oro province is known for its alluvial gold deposits and has been an important gold-mining centre. As is a naturally occurring element commonly found as an impurity in metal ores. The processing of gold with sulphide minerals that contain arsenopyrite and other complex As sulphide minerals results in arsenic containing emissions and effluents, which must be carefully considered (Robins and Jayaweera 1992). Numerous studies have evaluated As in the vicinity of mining sites (Hoang et al. 2021) to assess the natural origin (Ramos-Miras et al. 2014; Nanos et al. 2015). Based on the As levels noted in sediments, our objective was to evaluate if they could be transferred to shrimps and pose human health risks.

### PTE concentration in shrimps and the relation to biometric parameters

In general terms, the PTE concentration ranges in shrimps did not present very high values compared to the levels found in sediments and diet. The heavy metal contents in head (cephalothorax) and body (abdomen) of the shrimps collected from ponds are shown in Table 3. Heads contained significantly higher concentrations of all the PTEs (*p*<0.05) *versus* the body, except for Hg (higher in the body), and Cr showed similar concentrations in the head and the body (Table 3). The mean Cr value in the body was 1.09 mg kg^−1^ (range of 0.01–6.52 mg kg^−1^), which falls within the ranges noted for shrimps in Bangladesh with 0.24 mg kg^−1^ (Biswas et al. 2021) or 0.69 mg kg^−1^ (Sultana et al. 2022), and in Malaysia with 0.99 mg kg^−1^ (Lee et al. 2017) and Hong Kong with 2 mg kg^−1^ (Cheung and Wong 2006), but are disproportionate to the concentration of 20.86 mg kg^−1^ encountered in the shrimps farmed in Guangdong (China) (Wu and Yang 2011).

**Table 3:** Levels of PTE in shrimps head and body samples

			Cephalothorax					Abdomen (muscle)		
TPE	Min.	Max.	Median	Mean	SD	Min.	Max.	Median	Mean	SD
As	3.52	6.11	4.60	4.63^a^	0.65	0.91	3.21	1.99	2.02^b^	0.49
Cd	0.02	0.10	0.05	0.05^a^	0.02	0.01	0.02	0.01	0.01^b^	0.00
Co	0.14	0.49	0.28	0.28^a^	0.10	0.01	0.43	0.05	0.08^b^	0.10
Cr	0.23	4.89	0.73	1.00^a^	0.99	0.01	6.52	0.38	1.09^a^	1.70
Cu	99.98	233.01	141.02	145.74^a^	35.02	20.70	72.44	47.56	46.17^b^	12.13
Hg	10.00	68.81	31.69	32.52^a^	13.31	10.00	67.18	43.87	40.13^b^	16.18
Ni	0.52	1.86	0.82	0.88^a^	0.28	0.15	2.03	0.42	0.55^b^	0.44
Pb	0.24	1.09	0.69	0.64^a^	0.19	0.01	0.69	0.14	0.20^b^	0.17
Zn	51.80	100.51	73.41	72.92^a^	9.36	31.21	66.11	53.75	53.94^b^	6.55

PTE contents in mg kg^−1^ except Hg in μg kg^−1^

*Min*. minimum value, *Max*. maximum value, *Mean* mean value, *SD* standard deviation

*Different letter indicates statistically significant differences (*P* <0.05) after Kruskal–Wallis test, among head and body

There is high variability when comparing our level of PTEs to those in other studies. For example, Ni values in Ecuador lie at 0.15–2.03 mg kg^−1^ (mean 0.55 mg kg^−1^) and are similar to those indicated in Malaysia 0.45 mg kg^−1^ (Lee et al. 2017) or 0.05 mg kg^−1^ in Bangladesh (Biswas et al. 2021), but are much lower than the 400 mg kg^−1^ observed in the Hong Kong Nature Reserve (Cheung and Wong 2006). On the other hand, Cd content of most of the analysed shrimp in our study was below the analytical LoD (0.01 mg kg^−1^) in the body, was in accordance with the contents observed in other shrimp-farming areas, and was not detected on these farms in Malaysia (Lee et al. 2017) or in the Guangdong Province of China (Wu and Yang 2011), and its levels were low in shrimp-producing regions of Hong Kong (0.02 mg kg^−1^) (Cheung and Wong 2006) and Bangladesh (0.04 a 0.09 mg kg^−1^) (Biswas et al. 2021; Sultana et al. 2022). It was surprising to find on farms in Mexico that commercialise shrimps with Cd contents of 19.2 mg kg^−1^ (Páez-Osuna and Tron-Mayen 1996).

Other heavy metals to bear in mind on shrimp farms are Cu and Zn. These PTEs are essential trace elements that play a crucial role in the metabolism of crustaceans. Cu and Zn are present in the diet they are supplied with (Table 2), which means that these PTEs tend to accumulate in shrimp tissues (Arisekar et al. 2022; Aytekin et al. 2019; Páez-Osuna and Tron-Mayen 1996). The contents of Cu 46.17 (mg kg^−1^) and Zn 53.94 (mg kg^−1^) had the highest values in the PTEs evaluated in the body (Table 3). Compared to other studies, our Cu and Zn contents are higher than those for Malaysia (Cu 5.85 mg kg^−1^ and Zn 2.17 mg kg^−1^) (Lee et al. 2017) or Bangladesh (Cu 9.43 mg kg^−1^ and Zn 18.89 mg kg^−1^) (Sultana et al. 2022), and fall within the range for those observed in the Guangdong area of China (Cu 24.26 mg kg^−1^ and Zn 171.56 mg kg^−1^) (Wu and Yang 2011) or Bangladesh (Cu 31 mg kg^−1^ and Zn 43 mg kg^−1^) (Dietrich and Ayers 2021), but are lower than those in Hong Kong (Cu 110 mg kg^−1^ and Zn 90 mg kg^−1^) (Cheung and Wong 2006). Regarding non-essential elements like Pb and Hg with marked toxic capacity, we found that both Pb (0.20 mg kg^−1^) and Hg (40 μg kg^−1^) (Table 3) fell within the ranges found in other countries. The Pb contents in the body of the shrimps farmed in Bangladesh are approximately 0.50 mg kg^−1^ (Biswas et al. 2021; Dietrich and Ayers 2021), and 0.02 mg kg^−1^ in Malaysia, with a mean content of 10 mg kg^−1^ in Hong Kong (Cheung and Wong 2006). In the above-cited works, Hg presents less variability with about 20 μg kg^−1^ in Bangladesh (Sultana et al. 2022) or 70 μg kg^−1^ in Hong Kong (Cheung and Wong 2006), with a similar mean concentration to that obtained in the present study (mean of 40 μg kg^−1^). In any case, none of these PTEs herein analysed on shrimp farms in Ecuador presents higher contents than the limits set by European Commission (EuropeanCommission 2006), which sets maximum levels only for certain contaminants in foodstuff. Only our As levels, which went from 0.91 to 3.21 mg kg^−1^ in the body (mean of 2.02 mg kg^−1^), can be taken as really high. The decision is based on a 2021 scientific report from the European Food Safety Authority (EFSA) and EU rules follow the Codex Alimentarius maximum level of 0.5 mg/kg for total As. Our mean As contents are much higher than those detected in Bangladesh with 0.04 mg kg^−1^ (Sultana et al. 2022) or Malaysia with 0.46 mg kg^−1^ (Lee et al. 2017). However, we must highlight the mean values of 26.9 mg kg^−1^ found on shrimp farms in southern China (Wu et al. 2017).

**Fig. 1:**
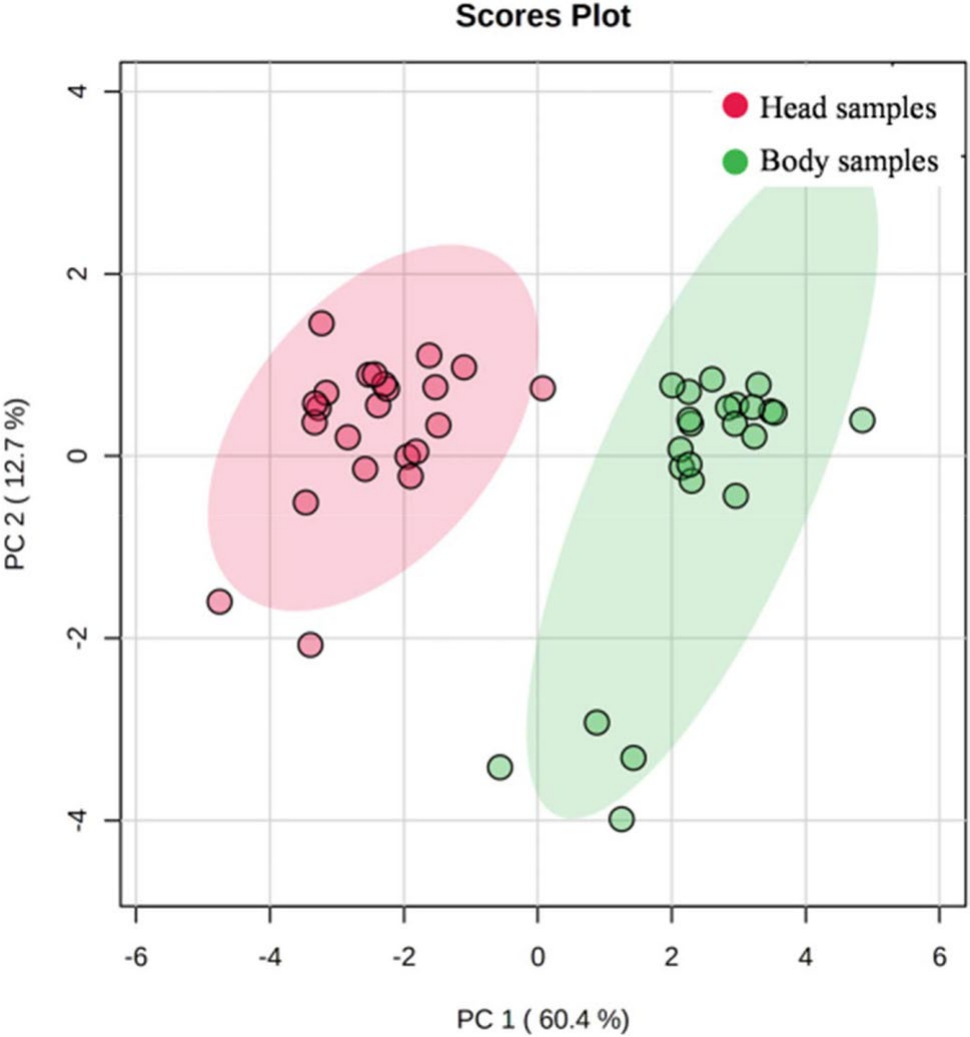
The principal component analysis for PTEs in shrimps according to the evaluated shrimp part (head vs body)

**Fig. 2:**
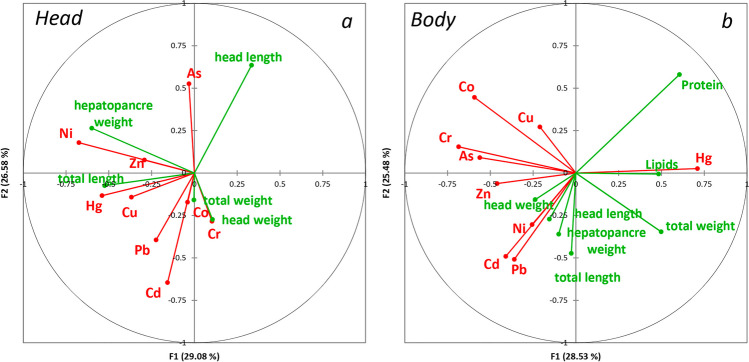
**a**, **b** Ordination diagram based on the canonical correlation analyses (CCorA) of PTE in head and body tissues versus the biometric indices and body composition parameters

In short, significantly higher As, Cd, Co, Cu, Ni, Pb and Zn concentrations were found in the cephalothorax of shrimp (Table 4). Only Hg presented higher concentrations in muscles than in the head (Table 3), a tendency that has also been observed in some marine fish species (Sánchez-Muros et al. 2018). Nonetheless, shrimps tend to accumulate more metals than fish, mainly in the head (gills, antennae and hepatopancreas) (Albuquerque et al. 2020; El-Said et al. 2021; Batvari et al. 2016). It was noted that viscera tended to accumulate higher concentrations of heavy metals than in muscle (Ramos-Miras et al. 2019; Lee et al. 2017). The principal component analysis (PCA) confirmed this situation (Fig. 1) and pointed out different behaviours for PTEs in shrimps in accordance with the evaluated body part. The tendency to accumulate PTEs is often associated with the species biometric parameters (Sánchez-Muros et al. 2018; Ramos-Miras et al. 2019; Sofoulaki et al. 2018; Renteria et al. 2022). From this point of view, we analysed the PTE relation in muscle and head tissues *versus* the biometric indices. Canonical correlation analyses (CCorA) were used to study these relations and were plotted separately for both (Fig. 2). In the cephalothorax (Fig. 2a), we observed how high As contents were associated with head weight, while the other heavy metals, including Hg, were related to hepatopancreas weight and total shrimp length. However, in the body (Fig. 2b), where Hg tends to accumulate, this heavy metal was significantly associated with the contents of proteins and lipids, and also with total shrimp weight, whereas all the other PTEs were related mostly to head weight, which was not statistically significant. This behaviour conditions the possible toxicity of shrimps as food.

**Table 4:** Statistic report on the multi-way analysis of variance of the tissues (head and body) and the diet of shrimp with the interaction of the main effects

Source	As	Cd	Co	Cr	Cu	Hg	Ni	Pb	Zn	Weight	Length
Diet	0.034	0.019	ns.	ns.	ns.	ns.	0.022	ns.	0.015	ns.	0.006
Tissue	***P*< 0.01**	***P*< 0.01**	***P*< 0.01**	***P*< 0.01**	***P*< 0.01**	***P*< 0.05**	***P*< 0.05**	***P*< 0.01**	***P*< 0.01**	ns.	ns.
Diet x Tissue	ns.	0.032	ns.	ns.	ns.	ns.	ns.	ns.	ns.	ns.	ns.

Differences between diet and tissue of Pacific White shrimp obtained by the ANOVA test.

Significant at 95% (*P* < 0.05) and 99% (*P* < 0.01). *ns* not significantly different

### Bioaccumulation factors (BSAF, BDAF) and target hazard quotient

**Fig. 3:**
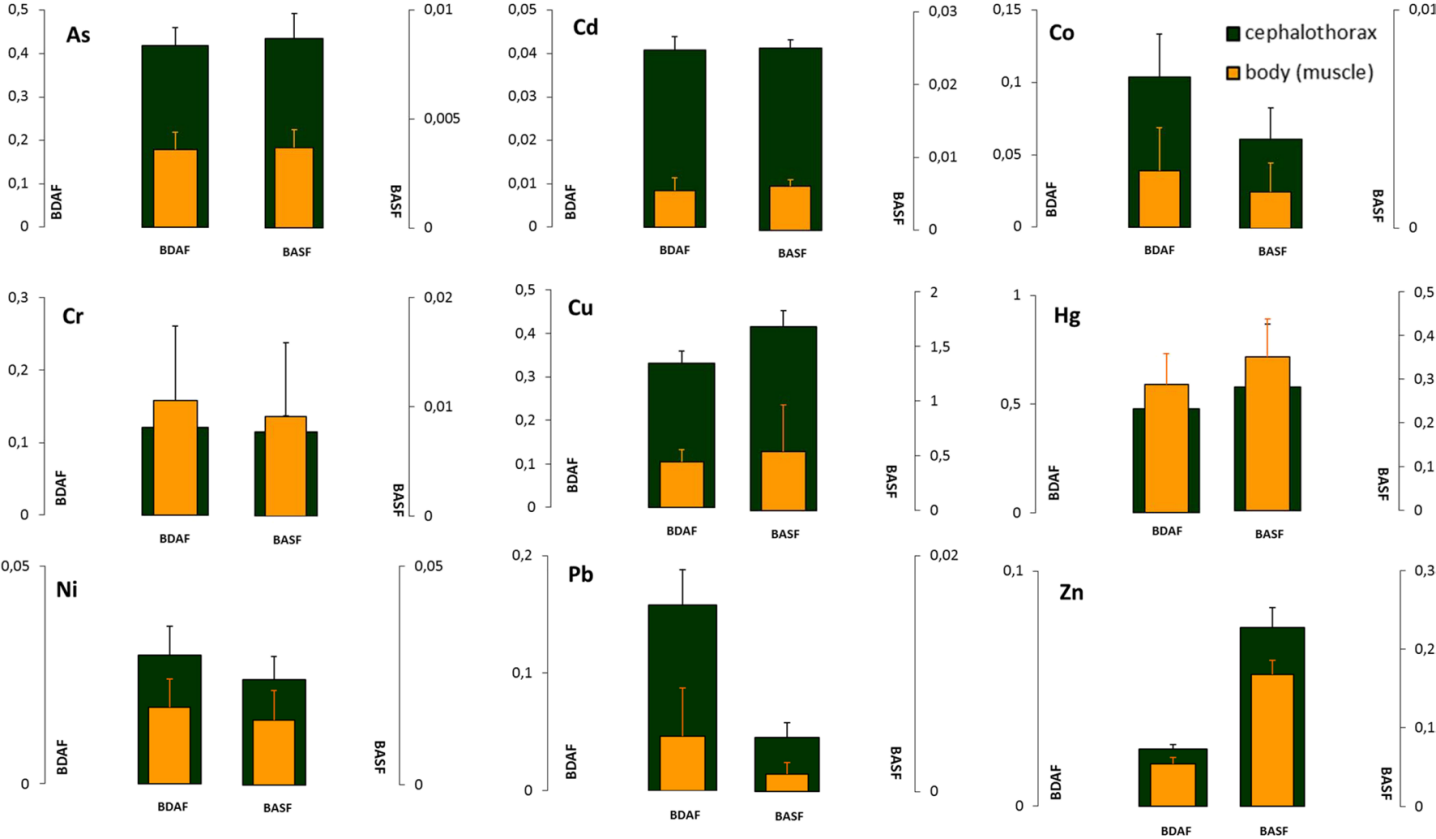
Bioaccumulation factors (BSAF, BDAF) for PTE in the head and the body of the studied shrimp in Ecuador

Heavy metals can accumulate in fish and shrimp tissues, which are generally found in the last zone of the aquatic food chain and have adverse effects on human health although these elements do not affect the biota. Crustaceans are able to accumulate metals at high body concentrations depending on the relation between the absorption and excretion of metals and the diluting body growth rate (Rainbow 2018), and can be transmitted through the food chain (Ali and Khan 2019). The PTE concentration in water and the food of aquatic species are important factors that affect PTE accumulation in aquatic organisms (Maceda-Veiga et al. 2012). Figure 3 and Table S3 show the BSAF and BDAF values, which were calculated for the head and the body of the studied shrimp in Ecuador. Except for Cu and Zn, the BADF value was higher in all cases than that of BASF, and no toxic effects were observed on the studied shrimp. This made us realise that the main factor of the influence on their accumulation might be diet. Nonetheless, it is not easy to determine if the metals that accumulate in shrimps come from diet or sediment because in semi-extensive shrimp farming, shrimps not only eat their diet, but also feed on the mixture of microorganisms that are produced in their ponds and on the remains of the organisms in these ponds. So, metals from the environment are incorporated into the shrimp food chain. However, as the levels of the heavy metals present in shrimp diet were much lower than those obtained in mud, except for Cu and Zn that are directly added to diet, their accumulation probably occurs directly from mud or indirectly through their incorporation into the pond’s microbiota that, in turn, forms part of shrimps’ intake. Indeed shrimps eat on carrion, which stems from a wide range of materials and include other animals living on the bottom of ponds and residue (Jiao et al. 2021).

We can consider that as no PTE bioaccumulation occurred with shrimps, all the BSAF and BADF values were lower than 1 for all elements (values greater than 1 would indicate bioaccumulation) except for Cu, which tends to accumulate in the cephalothorax (BADF = 1.6964). This aspect has been previously noted with shrimp farming (Prangnell et al. 2022), as in wild shrimps in the Persian Gulf (Dadar et al. 2014). However, this factor must be taken into account owing to the possible biomagnification of this metal in aquatic ecosystems (Griboff et al. 2020). Similarly, the values of both BSAF and BADF indices were significantly higher in the cephalothorax than in the body (Fig. 3), which limits the transfer of these PTEs during human consumption. The cephalothorax accommodates viscera, which generally present greater PTE accumulation than muscles or skeletal tissues (Albuquerque et al. 2020). Nevertheless, shrimps seem capable of limiting the concentration of contaminants in their organism by regulating their intestinal absorption and excretion (homeostasis) (Nascimento et al. 2016; Silva et al. 2016). Even other metals like Ni tend to accumulate in the exoskeleton (Páez-Osuna and Tron-Mayen 1996) and are periodically removed through moulting. Only Hg displayed a significant tendency to accumulate in the body. Figure 3 shows how both the BSAF and BADF accumulation indices are higher in the head than in the body, which contrasts with other studies indicating that hepatopancreas was the tissue where Hg mostly accumulated, followed by muscle and the exoskeleton (Ruelas-Inzunza et al. 2009). Such behaviour in the differing Hg distribution is reflected in the previous section (Fig. 2) by the lipid and protein contents in shrimps.

**Figure:**
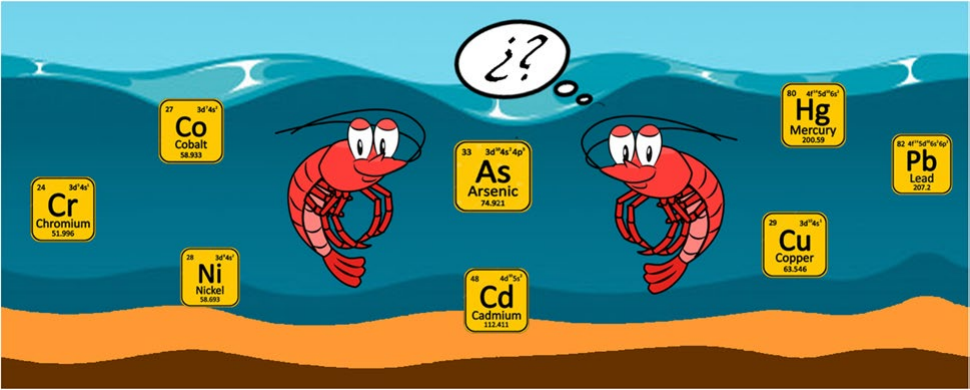
Table S4 contains the values obtained for the THQ and HI indices in edible shrimp parts. These indices are below 1 in all cases and THQ_Cu_ presents the highest values (16.99E^−3^ and 19.14E^−3^ for EU adults and US adults, respectively). Hence, there is no non-carcinogenic health risk for eating shrimp bodies for the different studied elements either alone (THQ) or combined (HI). There are not enough data available to compare the values of these indices because daily intakes and exposure times vary in shrimps among countries and their gastronomic habits. Some research (Wang et al. 2020; Yu et al. 2020) studied wild shrimps, while other research (Hidayati et al. 2020) studied shrimps from aquiculture. They obtained THQi and HI values below 1. Likewise, the values herein obtained are of the same order as those reported by (Sarkar et al. 2016) for shrimps farmed in SE Asia. Therefore, despite the population generally assuming that different shrimp species accumulate contaminants (Heidarieh et al. 2013), our data indicate that eating shrimps from Ecuador does not pose a health risk regarding the PTEs analysed in this study.

## Conclusions

Our results show that levels of PTE in water and sediment do not present a toxicity problem for animals in this aquatic environment, despite being an area of uncontrolled mining exploitations, except of As concentration in sediment, although its transfer to aquatic organisms appears to be limited. Furthermore, consumption of shrimps (*L. vanamei*) from Ecuador does not pose a consumer health risk because the levels of heavy metals are below the recommended limits, especially in muscles that are the edible part. Only As had a higher concentration than that set in the regulations applied in Europe, and only in the cephalothorax. Generally speaking, most of the PTEs tend to accumulate in the cephalothorax, which restricts them being transferred to human beings by intake and their health effects. Nonetheless, considerable discrepancy exists for the PTEs contents that appear in the bibliography, which sometimes exceed the recommended limits for humans. The fact that coherence is lacking among the regulations that set limits for these PTEs in each country or zone makes it difficult to have a single perception of contaminants being transferred to the human food chain because these shrimps are exported all over the world. We must bear in mind shrimps’ origin and where they have been farmed, the food they have eaten and possible sources of contamination that may affect shrimp farms. It is not easy for consumers to acquire all this information, but the present study has noted that the accumulation of these metals is conditioned by the levels of the metals obtained where shrimps are farmed, especially Zn and Cu that are provided in their diet, but also in their environment and in industrial activity, which affect such levels in shrimp-farming ponds.

### Supplementary information

ESM 1 (DOCX 14 kb)

ESM 2 (DOCX 13 kb)

ESM 3 (DOCX 15 kb)

ESM 4 (DOCX 15 kb)

## References

[CR1] Albuquerque FEA, Minervino AHH, Miranda M, Herrero-Latorre C, Júnior RAB, Oliveira FLC, Dias SR, Ortolani EL, Lopez-Alonso M (2020). Toxic and essential trace element concentrations in the freshwater shrimp Macrobrachium amazonicum in the Lower Amazon, Brazil. J Food Compos Anal.

[CR2] Ali H, Khan E (2019). Trophic transfer, bioaccumulation, and biomagnification of non-essential hazardous heavy metals and metalloids in food chains/webs—concepts and implications for wildlife and human health. Hum Ecol Risk Assess Int J.

[CR3] Appleton J, Williams T, Orbea H, Carrasco M (2001). Fluvial contamination associated with artisanal gold mining in the Ponce Enriquez, Portovelo-Zaruma and Nambija areas, Ecuador. Water Air Soil Pollut.

[CR4] Argandona LB (2016). Sector Camaronero: Evolución y proyección a corto plazo.

[CR5] Arisekar U, Shakila RJ, Shalini R, Jeyasekaran G, Padmavathy P, Hari MS, Sudhan C (2022). Accumulation potential of heavy metals at different growth stages of Pacific white leg shrimp, Penaeus vannamei farmed along the Southeast coast of Peninsular India: a report on ecotoxicology and human health risk assessment. Environ Res.

[CR6] Avigliano E, Monferrán MV, Sánchez S, Wunderlin DA, Gastaminza J, Volpedo AV (2019). Distribution and bioaccumulation of 12 trace elements in water, sediment and tissues of the main fishery from different environments of the La Plata basin (South America): Risk assessment for human consumption. Chemosphere.

[CR7] Aytekin T, Kargın D, Çoğun HY, Temiz Ö, Varkal HS, Kargın F (2019). Accumulation and health risk assessment of heavy metals in tissues of the shrimp and fish species from the Yumurtalik coast of Iskenderun Gulf, Turkey. Heliyon.

[CR8] Batvari BPD, Sivakumar S, Shanthi K, Lee K-J, Oh B-T, Krishnamoorthy R, Kamala-Kannan S (2016). Heavy metals accumulation in crab and shrimps from Pulicat lake, north Chennai coastal region, southeast coast of India. Toxicol Ind Health.

[CR9] Biswas C, Soma SS, Rohani MF, Rahman MH, Bashar A, Hossain MS (2021). Assessment of heavy metals in farmed shrimp, Penaeus monodon sampled from Khulna, Bangladesh: An inimical to food safety aspects. Heliyon.

[CR10] Caballero-Gallardo K, Alcala-Orozco M, Barraza-Quiroz D, De la Rosa J, Olivero-Verbel J (2020). Environmental risks associated with trace elements in sediments from Cartagena Bay, an industrialized site at the Caribbean. Chemosphere.

[CR11] Campos-Herrera R, El-Borai FE, Rodríguez Martín JA, Duncan LW (2016). Entomopathogenic nematode food web assemblages in Florida natural areas. Soil Biol Biochem.

[CR12] Cheung K-C, Wong MH (2006). Risk assessment of heavy metal contamination in shrimp farming in Mai Po Nature Reserve, Hong Kong. Environ Geochem Hlth.

[CR13] Copat C, Grasso A, Fiore M, Cristaldi A, Zuccarello P, Santo Signorelli S, Conti GO, Ferrante M (2018). Trace elements in seafood from the Mediterranean sea: an exposure risk assessment. Food Chem Toxicol.

[CR14] Dadar M, Peyghan R, Memari HR (2014). Evaluation of the bioaccumulation of heavy metals in white shrimp (Litopenaeus vannamei) along the Persian Gulf coast. B Environ Contam Tox.

[CR15] Dayal JS, Ponniah A, Khan HI, Babu EM, Ambasankar K, Vasagam KK (2013) Shrimps–a nutritional perspective. Curr Sci:1487–1491

[CR16] Dietrich M, Ayers J (2021). Geochemical partitioning and possible heavy metal (loid) bioaccumulation within aquaculture shrimp ponds. Sci Total Environ.

[CR17] EC-EuropeanCommission (2002). Directive (2002/32/EC) of the European Parliament and of the Council of 7 May 2002 on undesirable substances in animal feed. Off J Eur Union C.

[CR18] EC-EuropeanCommission (2015) Commission Regulation (EU) 2015/186 of 6 February 2015 amending Annex I to Directive 2002/32/EC of the European Parliament and of the Council as regards maximum levels for arsenic, fluorine, lead, mercury, endosulfan and Ambrosia seeds (Text with EEA relevance). Off J Eur Union

[CR19] El-Said GF, El-Sadaawy MM, Shobier AH, Ramadan SE (2021). Human health implication of major and trace elements present in commercial crustaceans of a traditional seafood marketing region, Egypt. Biol Trace Elem Res.

[CR20] Escobar-Segovia K, Jiménez-Oyola S, Garcés-León D, Paz-Barzola D, Navarrete EC, Romero-Crespo P, Salgado B (2021). Heavy metals in rivers affected by mining activities in Ecuador: pollution and human health implications. WIT Trans Ecol Environ.

[CR21] EUMOFA (2018). The EU fish market.

[CR22] EuropeanCommission E (2006). Commission Regulation (EC) No 1881/2006 of 19 December 2006 setting maximum levels for certain contaminants in foodstuffs. Off J Eur Union.

[CR23] Frías-Espericueta MG, Bautista-Covarrubias JC, Osuna-Martínez CC, Delgado-Alvarez C, Bojórquez C, Aguilar-Juárez M, Roos-Muñoz S, Osuna-López I, Páez-Osuna F (2022). Metals and oxidative stress in aquatic decapod crustaceans: a review with special reference to shrimp and crabs. Aquat Toxicol.

[CR24] García MÁ, Núñez R, Alonso J, Melgar MJ (2016). Total mercury in fresh and processed tuna marketed in Galicia (NW Spain) in relation to dietary exposure. Environ Sci Pollut R.

[CR25] Griboff J, Wunderlin DA, Horacek M, Monferrán MV (2020). Seasonal variations on trace element bioaccumulation and trophic transfer along a freshwater food chain in Argentina. Environ Sci Pollut R.

[CR26] Guhathakurta H, Kaviraj A (2000). Heavy metal concentration in water, sediment, shrimp (Penaeus monodon) and mullet (Liza parsia) in some brackish water ponds of Sunderban, India. Mar Pollut Bull.

[CR27] Heidarieh M, Maragheh MG, Shamami MA, Behgar M, Ziaei F, Akbari Z (2013). Evaluate of heavy metal concentration in shrimp (Penaeus semisulcatus) and crab (Portunus pelagicus) with INAA method. SpringerPlus.

[CR28] Hidayati NV, Prudent P, Asia L, Vassalo L, Torre F, Widowati I, Sabdono A, Syakti AD, Doumenq P (2020). Assessment of the ecological and human health risks from metals in shrimp aquaculture environments in Central Java, Indonesia. Environ Sci Pollut R.

[CR29] Hoang AT, Prinpreecha N, Kim K-W (2021). Influence of mining activities on arsenic concentration in rice in asia: A review. Minerals.

[CR30] Jiao Y, Yang L, Kong Z, Shao L, Wang G, Ren X, Liu Y (2021). Evaluation of trace metals and rare earth elements in mantis shrimp Oratosquilla oratoria collected from Shandong Province, China, and its potential risks to human health. Mar Pollut Bull.

[CR31] Jobson JD (1992). Applied multivariate data analysis.

[CR32] Lee WP, Payus C, Mohd Ali S, Vun LW (2017). Selected heavy metals in Penaeus vannamei (white prawn) in aquaculture pond near Likas Lagoon, Sabah, Malaysia. Int J Environ Sci Dev.

[CR33] León-Cañedo J, Alarcón-Silvas S, Fierro-Sañudo J, Mariscal-Lagarda M, Díaz-Valdés T, Páez-Osuna F (2017). Assessment of environmental loads of Cu and Zn from intensive inland shrimp aquaculture. Environ Monit Assess.

[CR34] Livingstone D (2001). Contaminant-stimulated reactive oxygen species production and oxidative damage in aquatic organisms. Mar Pollut Bull.

[CR35] Lopes RB, de Souza RF, Silva-Nicodemo SCT, Cruz JVF, de Medeiros GF (2018). Ecotoxicology of sediment in the estuary of the Jundiaí and Potengi Rivers in Natal-RN, Brazil, by using Leptocheirus plumulosus as test-organism. Ecotoxicol Environ Contam.

[CR36] Losi V, Ferrero TJ, Moreno M, Gaozza L, Rovere A, Firpo M, Marques JC, Albertelli G (2013). The use of nematodes in assessing ecological conditions in shallow waters surrounding a Mediterranean harbour facility. Estuar Coast Shelf Sci.

[CR37] Łuczyńska J, Paszczyk B, Łuczyński MJ (2018). Fish as a bioindicator of heavy metals pollution in aquatic ecosystem of Pluszne Lake, Poland, and risk assessment for consumer's health. Ecotoxicol Environ Saf.

[CR38] MacDonald DD, Ingersoll CG, Berger T (2000). Development and evaluation of consensus-based sediment quality guidelines for freshwater ecosystems. Arch Environ Contam Toxicol.

[CR39] Maceda-Veiga A, Monroy M, de Sostoa A (2012). Metal bioaccumulation in the Mediterranean barbel (Barbus meridionalis) in a Mediterranean River receiving effluents from urban and industrial wastewater treatment plants. Ecotoxicol Environ Saf.

[CR40] Mackay D, Celsie AK, Powell DE, Parnis JM (2018). Bioconcentration, bioaccumulation, biomagnification and trophic magnification: a modelling perspective. Environ Sci: Processes Impacts.

[CR41] Mestanza-Ramón C, Cuenca-Cumbicus J, D’Orio G, Flores-Toala J, Segovia-Cáceres S, Bonilla-Bonilla A, Straface S (2022). Gold mining in the Amazon Region of ecuador: history and a review of its socio-environmental impacts. Land.

[CR42] Mirzaei R, Ghorbani H, Hafezi Moghaddas N, Martín JAR (2014). Ecological risk of heavy metal hotspots in topsoils in the Province of Golestan, Iran. J Geochem Explor.

[CR43] Monsalve ER, Quiroga E (2022) Farmed shrimp aquaculture in coastal wetlands of Latin America—a review of environmental issues. Mar Pollut Bull:113956 10.1016/j.marpolbul.2022.113956 36058720

[CR44] Nanos N, Grigoratos T, Rodríguez Martín J, Samara C (2015). Scale-dependent correlations between soil heavy metals and As around four coal-fired power plants of northern Greece. Stoch Env Res Risk A.

[CR45] Nascimento JR, Bidone ED, Rolao-Araripe D, Keunecke KA, Sabadini-Santos E (2016). Trace metal distribution in white shrimp (Litopenaeus schmitti) tissues from a Brazilian coastal area. Environ Earth Sci.

[CR46] NationalMarineFisheriesService (2017) Fisheries of the United States. In: Yencho. MLM (ed)

[CR47] Odumo B, Carbonell G, Angeyo H, Patel J, Torrijos M, Rodríguez Martín J (2014). Impact of gold mining associated with mercury contamination in soil, biota sediments and tailings in Kenya. Environ Sci Pollut R.

[CR48] Odumo B, Nanos N, Carbonell G, Torrijos M, Patel JP, Rodríguez Martín JA (2018). Artisanal gold mining in a rural environment: land degradation in Kenya. Land Degrad Dev.

[CR49] Páez-Osuna F (2001). The environmental impact of shrimp aquaculture: a global perspective. Environ Pollut.

[CR50] Páez-Osuna F, Tron-Mayen L (1996). Concentration and distribution of heavy metals in tissues of wild and farmed shrimp Penaeus vannamei from the northwest coast of Mexico. Environ Int.

[CR51] Prangnell DI, Castro LF, Ali AS, Browdy CL, Samocha TM (2022). The performance of juvenile Litopenaeus vannamei fed commercial diets of differing protein content, in a super-intensive biofloc-dominated system. J Appl Aquac.

[CR52] Rainbow P (2018). Heavy metal levels in marine invertebrates. Heavy metals in the marine environment.

[CR53] Ramírez J, Lacasaña M (2001). Plaguicidas: clasificación, uso, toxicología y medición de la exposición. Arch Prev Riesgos Labor.

[CR54] Ramos-Miras JJ, Díaz-Férnandez P, SanJosé-Wery A, Rodríguez-Martin JA, Roca N, Bech J, Roca-Perez L, Boluda R, Gil C (2014) Influence of parent material and soil use on arsenic forms in soils: a case study in the Amblés Valley (Castilla-León, Spain). J Geochem Explor 147 (0):260-267. 10.1016/j.gexplo.2014.09.003

[CR55] Ramos-Miras JJ, Sanchez-Muros MJ, Morote E, Torrijos M, Gil C, Zamani-Ahmadmahmoodi R, Rodríguez Martin JA (2019). Potentially toxic elements in commonly consumed fish species from the western Mediterranean Sea (Almería Bay): bioaccumulation in liver and muscle tissues in relation to biometric parameters. Sci Total Environ.

[CR56] Renteria P, Vizcaíno AJ, Sánchez-Muros MJ, Santacruz-Reyes RA, Saez MI, Fabrikov D, Barroso FG, Vargas-García MC (2022). Effect of replacing fishmeal with Plukenetia volubilis cake on growth, digestive enzymes, and body composition in whiteleg shrimp (Litopenaeus vannamei). Fishes.

[CR57] Robins R, Jayaweera LD (1992). Arsenic in gold processing. Miner Process Extr Metall Rev.

[CR58] Rodríguez Martín JA, Gutiérrez C, Escuer M, García-González MT, Campos-Herrera R, Águila N (2014). Effect of mine tailing on the spatial variability of soil nematodes from lead pollution in La Union (Spain). Sci Total Environ.

[CR59] Ruelas-Inzunza J, Páez-Osuna F, Zamora-Arellano N, Amezcua-Martínez F, Bojórquez-Leyva H (2009). Mercury in biota and surficial sediments from Coatzacoalcos Estuary, Gulf of Mexico: distribution and seasonal variation. Water Air Soil Pollut.

[CR60] Sánchez-Muros MJ, Morote E, Gil C, Ramos-Miras JJ, Torrijos M, Rodríguez Martin JA (2018). Mercury contents in relation to biometrics and proximal composition and nutritional levels of fish eaten from the Western Mediterranean Sea (Almería bay). Mar Pollut Bull.

[CR61] Sarkar T, Alam MM, Parvin N, Fardous Z, Chowdhury AZ, Hossain S, Haque M, Biswas N (2016). Assessment of heavy metals contamination and human health risk in shrimp collected from different farms and rivers at Khulna-Satkhira region, Bangladesh. Toxicol Rep.

[CR62] Silva BM, Morales GP, Gutjahr AL, Freitas Faial KD, Carneiro BS (2018). Bioacumulation of trace elements in the crab Ucides cordatus (Linnaeus, 1763) from the macrotidal mangrove coast region of the Brazilian Amazon. Environ Monit Assess.

[CR63] Silva E, Viana Z, Onofre C, Korn M, Santos V (2016). Distribution of trace elements in tissues of shrimp species Litopenaeus vannamei (Boone, 1931) from Bahia, Brazil. Braz J Biol.

[CR64] Sofoulaki K, Kalantzi I, Machias A, Mastoraki M, Chatzifotis S, Mylona K, Pergantis SA, Tsapakis M (2018). Metals and elements in sardine and anchovy: species specific differences and correlations with proximate composition and size. Sci Total Environ.

[CR65] Sultana S, Hossain MB, Choudhury TR, Yu J, Rana MS, Noman MA, Hosen MM, Paray BA, Arai T (2022). Ecological and human health risk assessment of heavy metals in cultured shrimp and aquaculture sludge. Toxics.

[CR66] Takoutsing B, Martín JAR, Weber JC, Shepherd K, Sila A, Tondoh J (2017). Landscape approach to assess key soil functional properties in the highlands of Cameroon: repercussions of spatial relationships for land management interventions. J Geochem Explor.

[CR67] Takoutsing B, Weber JC, Martín JAR, Shepherd K, Aynekulu E, Sila A (2018). An assessment of the variation of soil properties with landscape attributes in the highlands of Cameroon. Land Degrad Dev.

[CR68] Traina A, Bono G, Bonsignore M, Falco F, Giuga M, Quinci EM, Vitale S, Sprovieri M (2019). Heavy metals concentrations in some commercially key species from Sicilian coasts (Mediterranean Sea): potential human health risk estimation. Ecotoxicol Environ Saf.

[CR69] USEPA U (2000). Risk-based concentration table.

[CR70] Uzcátegui C, Solano J, Figueroa P (2016). Perspectiva sobre la sostenibilidad de los recursos naturales a largo plazo: caso industria camaronera ecuatoriana. Revista Universidad y Sociedad.

[CR71] Vilela-Pincay W, Espinosa-Encarnación M, Bravo-González A (2020). La contaminación ambiental ocasionada por la minería en la provincia de El Oro. Estudios de la Gestión: revista internacional de administración.

[CR72] Walpole SC, Prieto-Merino D, Edwards P, Cleland J, Stevens G, Roberts I (2012). The weight of nations: an estimation of adult human biomass. BMC Public Health.

[CR73] Wang X, Wu J, Yu B, Dong KF, Ma D, Xiao G, Zhang C (2020). Heavy metals in aquatic products and the health risk assessment to population in China. Environ Sci Pollut R.

[CR74] Wu H, Liu J, Bi X, Lin G, Feng CC, Li Z, Qi F, Zheng T, Xie L (2017). Trace metals in sediments and benthic animals from aquaculture ponds near a mangrove wetland in Southern China. Mar Pollut Bull.

[CR75] Wu X-Y, Yang Y-F (2011). Heavy metal (Pb, Co, Cd, Cr, Cu, Fe, Mn and Zn) concentrations in harvest-size white shrimp Litopenaeus vannamei tissues from aquaculture and wild source. J Food Compos Anal.

[CR76] Xu X, Huo Q, Dong Y, Zhang S, Yang Z, Xian J, Yang Y, Cheng Z (2019). Bioaccumulation and health risk assessment of trace metals in fish from freshwater polyculture ponds in Chengdu, China. Environ Sci Pollut R.

[CR77] Yu B, Wang X, Dong KF, Xiao G, Ma D (2020). Heavy metal concentrations in aquatic organisms (fishes, shrimp and crabs) and health risk assessment in China. Mar Pollut Bull.

[CR78] Zamani-Ahmadmahmoodi R, Gharahi N, Rodríguez Martin JA, Aazami J, Jafari A, Bahmani M, Jiménez-Ballesta R (2020). Cd and Pb bioaccumulation in Eurasian watermilfoil (Myriophyllum spicatum) in relation to the role of metal contents in wetland sediments. Environ Monit Assess.

