# Supplementary material for: Potentially toxic element bioaccumulation in consumed indoor shrimp farming associated with diet, water and sediment levels
Source: Environ Sci Pollut Res Int. 2023 Nov 14;30(58):121794–806. doi: 10.1007/s11356-023-30939-1 (PMC10724093; doi:10.1007/s11356-023-30939-1)
Supplement: Supplementary file 1 — (DOCX 14 kb) [file 11356_2023_30939_MOESM1_ESM.docx]

Table S1: Shrimp biometric parameters

| Total weight | 14.33604 | ± | 2.3613 |
| --- | --- | --- | --- |
| Cephalothorax weight | 3.98517 | ± | 0.5933 |
| Abdomen weigt | 8.74226 | ± | 1.174 |
| Abdomen weight + exoskeleton | 10.20478 | ± | 1.7663 |
| Hepatopacreas weight | 0.41748 | ± | 0.1288 |
| Total leght | 12.758 | ± | 1.4011 |
| Cephalothorax lenght | 5.65 | ± | 0.2991 |
| Abdomen lenght | 8.96 | ± | 0.6096 |
| SGR (%) | 2.06527489 | ± | 0.0343 |
| DAILY GAIN (g/day) | 0.16525714 | ± | 0.0134 |
| FCR | 0.93851007 | ± | 0.0773 |
