# Supplementary material for: Potentially toxic element bioaccumulation in consumed indoor shrimp farming associated with diet, water and sediment levels
Source: Environ Sci Pollut Res Int. 2023 Nov 14;30(58):121794–806. doi: 10.1007/s11356-023-30939-1 (PMC10724093; doi:10.1007/s11356-023-30939-1)
Supplement: Supplementary file 2 — (DOCX 13 kb) [file 11356_2023_30939_MOESM2_ESM.docx]

Table S1: Sediment Quality Guidelines concentration (mg kg^-1^, dw)

|  | **As** | **Cd** | **Cr** | **Cu** | **Hg** | **Pb** |
| --- | --- | --- | --- | --- | --- | --- |
| TEL: Threshold Effects Level | 7.2 | 0.7 | 52.3 | 18.7 | 0.174 | 30.2 |
| PEL: Probable Effect Level | 41.6 | 4.2 | 160.4 | 108.2 | 0.486 | 112.2 |
| ERM: Effects Range Medium | 70 | 9.6 | 370 | 270 | 0.71 | 218 |
