# Supplementary material for: Potentially toxic element bioaccumulation in consumed indoor shrimp farming associated with diet, water and sediment levels
Source: Environ Sci Pollut Res Int. 2023 Nov 14;30(58):121794–806. doi: 10.1007/s11356-023-30939-1 (PMC10724093; doi:10.1007/s11356-023-30939-1)
Supplement: Supplementary file 3 — (DOCX 15 kb) [file 11356_2023_30939_MOESM3_ESM.docx]

Table 5: Target hazard quotient (THQ) and Total Target hazard quotient (TTHQ) for PTE in adults

|  | **European Union adults** | | | | | | | | | |
| --- | --- | --- | --- | --- | --- | --- | --- | --- | --- | --- |
|  | **THQ As** | **THQ Cd** | **THQ Co** | **THQ Cr** | **THQ Cu** | **THQ Hg** | **THQ Ni** | **THQ Pb** | **THQ Zn** | **TTHQ** |
| Min. | 1.45E-3 | 1.15 E-4 | 0.02 E-4 | 0.02E-5 | 7.52 E-3 | 2.24 E-3 | 0.11 E-3 | 0.66E-4 | 1.51 E-3 | 0.014 |
| Mean | 2.97E-3 | 1.80 E-4 | 0.25E-4 | 0.12E-4 | 16.99 E-3 | 4.06 E-3 | 0.41 E-3 | 8.42E-4 | 2.65 E-3 | **0.031** |
| Max. | 5.01E-3 | 2.62 E-4 | 1.14E-4 | 0.69E-4 | 24.75 E-3 | 5.5 E-3 | 1.59 E-3 | 27.30E-4 | 3.44 E-3 | 0.041 |
| SD | 0.80E-3 | 0.55 E-4 | 0.31E-4 | 0.19E-4 | 4.47 E-3 | 0.94 E-3 | 0.37 E-3 | 6.54E-4 | 0.38 E-3 | 0.006 |
|  | **United Stated of America adults** | | | | | | | | | |
|  | **THQ As** | **THQ Cd** | **THQ Co** | **THQ Cr** | **THQ Cu** | **THQ Hg** | **THQ Ni** | **THQ Pb** | **THQ Zn** | **HI** |
| Min. | 1.63E-3 | 1.30 E-4 | 0.02 E-4 | 0.02E-5 | 8.46 E-3 | 2.52 E-3 | 0.13 E-3 | 0.74 E-4 | 1.70 E-3 | 0.017 |
| Mean | 3.35 E-3 | 2.03 E-4 | 0.29 E-4 | 0.14 E-4 | 19.14 E-3 | 4.57 E-3 | 0.46E-3 | 9.48 E-4 | 2.98 E-3 | **0.041** |
| Max. | 5.65 E-3 | 2.95 E-4 | 1.28 E-4 | 0.78 E-4 | 27.86 E-3 | 6.29 E-3 | 1.79 E-3 | 30.74 E-4 | 3.87 E-3 | 0.064 |
| SD | 0.90 E-3 | 0.61 E-4 | 0.35 E-4 | 0.21 E-4 | 5.04 E-3 | 1.06 E-3 | 0.41 E-3 | 7.36 E-4 | 0.43 E-3 | 0.010 |

Min.-minimum value. Mean-mean value. Max.-maximum value. SD-standard deviation.
